# Gender difference in the association between gout at diagnosis and metabolic syndrome in African population: a retrospective cohort study

**DOI:** 10.11604/pamj.2022.43.164.37197

**Published:** 2022-12-01

**Authors:** Fernando Kemta Lekpa, Francine Same Bebey, Isabel Bouallo, Sylvain Raoul Simeni Njonnou, Henry Namme Luma, Madeleine Singwe-Ngandeu, Simeon-Pierre Choukem

**Affiliations:** 1 Rheumatology Unit, Department of Internal Medicine, Douala General Hospital, Douala, Cameroon,; 2 Department of Internal Medicine and Specialties, Faculty of Medicine and Pharmaceutical Sciences, University of Dschang, Dschang, Cameroon,; 3 Health and Human Development Research Group, Douala, Cameroon,; 4 Department of Clinical Sciences, Faculty of Medicine and Pharmaceutical Sciences, University of Douala, Douala, Cameroon,; 5 Department of Internal medicine and subspecialties, Faculty of Medicine and Biomedical Sciences, University of Yaoundé I, Yaoundé, Cameroon,; 6 Department of Rheumatology, Yaoundé Central Hospital, Yaoundé, Cameroon

**Keywords:** Gout, hyperuricemia, metabolic syndrome, female

## Abstract

**Introduction:**

few studies have specifically investigated the link between metabolic syndrome (MetS) and gout in sub-Saharan Africa. This report aimed to evaluate in patients with gout the association between gout at diagnosis and MetS and to assess the gender difference.

**Methods:**

we performed a single-centre retrospective cohort study on all outpatients seen in the Rheumatology Unit of the General Hospital, Douala, Cameroon. We included records of patients with a recent diagnosis of gout according to the American College of Radiology (ACR) criteria. MetS was defined according to the harmonized criteria.

**Results:**

we included 511 patients (415 men), with a mean age at diagnosis of 55.9 ±10.8 years. Women were older than men. The mean serum uric acid was 8.24±2.23 mg/L, with hyperuricemia found in 394 patients (77.1%). MetS was present in 101 patients (19.7% [95% CI: 16.8%-22.1%]), significantly more common in men compared to women (23.6% vs. 10.8%; p<0.001). The main components of the MetS were: increased waist circumference (217 patients, 61.3%), obesity (256 patients, 52.2%), hypertension (208 patients, 40.7%), and diabetes mellitus (52 patients, 10.2%). Furthermore, hypertension, diabetes mellitus, obesity, and increased waist circumference were more frequent in women (p<0.001). There was no difference in dyslipidemia according to gender. The combination of components of the MetS was more frequent in men than women (p<0.001).

**Conclusion:**

MetS are common in newly diagnosed Cameroonian patients with gout, with increased waist circumference, obesity, hypertension and diabetes mellitus being the main components. These components are more common in women, but their combination was more frequent in men.

## Introduction

Metabolic syndrome (MetS) is a set of morphologic, physiologic, and biochemical conditions that can coexist together and increase the risk of type 2 diabetes and atherosclerotic heart disease [1]. MetS shares with gout and its necessary precursor hyperuricemia, the same traditional cardiovascular risk factors including hypertension, insulin resistance, type 2 diabetes, atherosclerotic heart disease, obesity, dyslipidemia, and chronic kidney disease [1-4]. This relationship between hyperuricemia/gout and MetS remains controversial. However, increasing evidence shows that although hyperuricemia is more common in men, it is a strong and independent predictor of incident MetS in both men and women [1,5,6]. The main pathophysiological links between hyperuricemia and the MetS are insulin resistance, renal urate under-excretion, urate-induced systemic inflammation, and a novel condition termed non-alcoholic fatty kidney disease [7-9]. In addition, evidence shows that the difference in risk of cardiovascular disease by gender in patients without gout disappears in men and women with gout. Gout-induced systemic inflammation in women, who otherwise have a lower prevalence of cardiac risk factors than age-matched men, is more atherogenic than in men [10]. All these arguments have led some authors to propose the inclusion of hyperuricemia and gout among the components of MetS [1]. The overall prevalence of MetS is high, affecting 11.1%-23.9% of the general population in sub-Saharan Africa (SSA) according to diagnostic criteria used [11]. In addition, the prevalence of MetS was higher in women than in men [11]. In Cameroon, this prevalence varies from 25% to 32.4%, also with a clear female predominance [12,13]. In this country, about one out of every two gout patients would have a MetS [14]. These patients with gout and MetS significantly have a higher body mass index (BMI) and higher levels of serum uric acid (SUA). Although MetS was more common in women than in men, the difference was not significant [14]. These results are comparable to those obtained out of Africa [1]. However, the prevalence of MetS in patients with gout under real-life conditions has not been evaluated in Africans. Thus, we performed this study with the aim to determine the prevalence of MetS at the time of gout diagnosis in a hospital setting in SSA. Also, the particularities related to gender will be evaluated.

## Methods

**Study design and setting:** we conducted a single-centre retrospective cohort study from January 2004 to December 2014 at of the Douala General Hospital. This hospital is a tertiary and teaching hospital located in Douala, the economic capital of Cameroon, Central Africa, with a capacity of 300 beds that receives patients from all the ten regions of Cameroun and neighboring countries. The study was performed at the outpatient consultations of the Rheumatology unit.

**Participants:** our study population consisted of all consenting consecutive outpatients who presented with the diagnosis of gout. In these patients with gout, we looked for components of the MetS. The following operational definitions have been adopted.

**Gout** was defined according to the American College of Rheumatology preliminary criteria [15]. Six of 12 clinical criteria are required, or the presence of monosodium urate (MSU) crystals in synovial fluid or tophus. Clinical criteria included: more than one attack of acute arthritis, maximum inflammation developed within one day, oligoarthritis attack, redness observed over joints, first metatarsophalangeal joint painful or swollen, unilateral first metatarsophalangeal joint attack, unilateral tarsal joint attack, tophus, hyperuricemia, asymmetric swelling within a joint on X-ray, subcortical cyst without erosions on X-ray, complete termination of an attack [15].

**Hyperuricemia** was defined as SUA level greater than 6.0 mg/dL [16].

**Metabolic syndrome (MetS)** was defined according to the ‘harmonized´ criteria set established by a joint scientific multisocietal committee in 2009 [17]. The diagnosis of MetS was retained if the patient met three or more of the following 5 criteria: 1) abdominal obesity (increased waist circumference greater than 94 cm in men and 80 cm in women; waist circumference thresholds defined according to the International Diabetes Federation cut points for sub-Saharan African), 2) elevated triglyceride (≥ 150 mg/dL; drug treatment for elevated triglycerides is an alternate indicator), 3) low high-density lipoprotein (HDL) cholesterol level (< 40 mg/dL in men and < 50 mg/dL in women; drug treatment for reduced HDL cholesterol is an alternate indicator), 4) elevated blood pressure (systolic ≥ 130 and/or diastolic ≥ 85 mmHg; antihypertensive drug treatment in a patient with a history of hypertension is an alternate indicator), and 5) impaired fasting glucose (≥ 100 mg/dL; drug treatment of elevated glucose is an alternate indicator) [17].

**Variables and data sources:** for each patient, data at the time of diagnosis of gout were collected on a standardized case-report form that included sociodemographic data (age, sex, place of abode), current and past medical history of the patient (with a special interest in the components of the MetS: obesity, hypertension, diabetes mellitus), clinical (arthralgia, synovitis, tophus, BMI, abdominal obesity), laboratory (SUA, triglyceride, cholesterol, fasting glucose), and therapeutic (particularly diuretics) data at the time of diagnosis.

**Bias:** when information was missing from the case-report form, the patient was contacted if he/she had a valid telephone number to obtain additional information. The components and purpose of the study were explained to these patients, and only those who freely gave written consent were included.

**Study size:** we used a consecutive, non-probability sampling method to select eligible study participants. We excluded pregnant women, patients with chronic kidney disease and patients on cancer and/or tuberculosis chemotherapy, as reported in their health records.

**Data management and statistical methods:** the data were collected and analyzed using SPSS version 22.0 software (Chicago, IL, USA). Quantitative variables were summarized and presented as mean ± standard deviation (SD). Qualitative variables were summarized using absolute numbers and percentages (%). Statistical comparisons were made with Student´s t-test for continuous variables and the Chi-square test for categorical variables. A p-value less than 0.05 was considered statistically significant.

**Ethical considerations:** this study received Ethical Approval from the Institutional Review Board. The requirement for written informed consent was waived for patient records without missing data because this study was analyzed retrospectively. Patient confidentiality was maintained, and the study adhered to the World Medical Association´s Declaration of Helsinki.

## Results

**General characteristics of patients with gout at diagnosis:** we included 511 patients (415 men and 96 women) aged 26 to 86 years. The mean age at the time of diagnosis was 55.9 ± 10.8 years. Women were older than men (62.56 ± 11.02 years vs. 54.45 ± 10.22 years; p < 0.001). The mean SUA level was 8.24 ± 2.23mg/dL, with hyperuricemia found in 394 patients (77.1%). Table 1 summarizes the main baseline characteristics of the study population.

**Table 1:** general characteristics of patients

Variable	All patients (n=511)	Sex	
		Men	Women
Age, mean ± SD years	55.9 ± 10.8	54.45 ± 10.22	62.56 ± 11.02
Sex, n (%)	511	415 (81.2%)	96 (18.8%)
BMI ≥ 25kg/m^2^, n (%)	321 (62.8%)	260 (50.9%)	61 (11.9%)
Diuretics use, n (%)	51 (9.9%)	36 (7.0%)	15 (2.9%)
Serum uric acid, mean ± SD, mg/dL	8.24 ± 2.23	8.3 ± 1.08	8.0 ± 2.71
Hyperuricemia*, n (%)	394 (77.1%)	329 (64.4%)	65 (12.7%)
Tophus, n (%)	203 (39.7%)	193 (37.8%)	10 (1.9%)
Arthralgia, n (%)	508 (99.4%)	414 (81.0%)	94 (18.4%)
Synovitis, n (%)	198 (38.7%)	172 (33.6%)	26 (5.1%)
MSU crystals (synovial fluid), n (%)^†^	22 (47.8%)	14 (63.6%)	8 (36.4%)
Urate arthropathies, n (%)^ǂ^	68 (28.1%)	58 (24.0%)	10 (4.1%)

* Hyperuricemia defined as SUA level > 7.0 mg/d; ^†^analysis of joint fluid was performed in 68 patients out of 87 patients with joint effusion. Monosodium urate (MSU) crystals were present in 22 patients (47.8%) of synovial fluid analyzed; ^ǂ^radiograph of the involved joint was done in 242 patients; SD: standard deviation

**Frequency of metabolic syndrome components:** the main components of the MetS were increased waist circumference (217 patients, 61.3%), obesity (256 patients, 52.2%), hypertension (208 patients, 40.7%), and diabetes mellitus (52 patients, 10.2%). Furthermore, hypertension, diabetes mellitus, obesity, and increased waist circumference were more frequent in women (p < 0.001). There was no difference in dyslipidemia according to gender (Table 2). According to the “harmonized” criteria set definition [17], MetS was present in 101 patients (19.7% [95% CI: 16.8%-22.1%]). It was significantly more common in men (23.6% [95% CI: 21.8%-25.2%]) compared to women (10.8% [95% CI: 6.1%-13.5%]); p < 0.001.

**Table 2:** association between gout and the components of metabolic syndrome

Variables*	Number of patients (%)	Sex		P-value^ǂ^
		Men (n = 415)	Women (n = 96)	
Obesity^†^; n = 490	256 (52.2%)	175 (42.2%)	81 (84.4%)	<0.001
Increased waist circumference; n = 143	89 (62.2%)	63 (15.2%)	26 (27.1%)	<0.001
Hypertension; n = 511	208 (40.7%)	155 (37.3%)	53 (55.2%)	<0.001
Diabetes mellitus; n = 511	79 (15.4%)	58 (13.9%)	21 (21.8%)	<0.001
Dyslipidemia; n = 104	70 (67.3%)	54 (13.0%)	16 (16.7%)	0.323

* Number of patients in whom the variable was filled; ^†^defined according to the body mass index (BMI≥25kg/m^2^); ^ǂ^Comparison between men and women

**Association between metabolic syndrome and with gender:** as the number of MetS components increased, the number of patients reduced (Table 3). The combination of components of the MetS was more frequent in men than women (p < 0.001).

**Table 3:** association between gout and the number of components of the metabolic syndrome

Number of component of MetS*	Number of patients (%)	Sex		P-value^†^
		Men	Women	
1 component	208 (40.7%)	145 (28.3%)	63 (12.4%)	0.178
2 components	89 (17.4%)	65 (12.7%)	24 (4.7%)	<0.001
3 components	86 (16.8%)	63(12.3%)	23(4.5%)	<0.001
4 components	38 (7.4%)	30(5.8%)	8(1.6%)	<0.001
5 components	7 (1.3%)	6(1.1%)	1(0.2%)	<0.001

* Metabolic syndrome, according to the harmonized criteria for clinical diagnosis (17); ^†^Comparison between men and women

## Discussion

This study aimed to evaluate the prevalence of MetS and its components in newly diagnosed patients with gout in Cameroon and seek an association with gender. Our study´s first result is that MetS is common in Cameroonian patients with gout at the time of diagnosis, where it concerns about 1 in 5 patients. Although it is low compared to the high prevalence previously reported in Cameroon [14] and elsewhere [1,18-25], these findings indicate that the prevalence of the MetS increases substantially with the duration of the disease, increasing levels of SUA, and even with age [1,18,20]. As a reminder, data were collected in this study at the time of diagnosis, whereas in the previous study performed in Cameroon the data were collected in patients with gout with a median duration of disease at 7.5 years [14]. Over time, the pro-oxidative, pro-inflammatory, and vasoconstrictive effects of SUA that may contribute to cardiometabolic diseases are expected to intensify and lead to an increased incidence of MetS [26]. Second, MetS was twice as common in men as in women with gout. The data available in the literature are discordant regarding gender. Indeed, male predominance is also found by some authors [5,22,27]. Other studies have found any difference between men and women [1,14]. However, when stratified by SUA level and gender, the prevalence of MetS in women tends to be higher than in men for a given category of SUA level of 6 mg/dL or more [18]. This may be justified by the fact that women with gout have a greater risk of incident cardiovascular events, despite a higher prevalence of both gout and cardiovascular disease in men [23]. Also, the difference in prevalence between men and women tends to disappear over time. This is the finding of a prospective study with a mean follow-up of 5.7 years, which shows that women with gout have an increased risk of developing MetS at least twice that of men with gout [5]. Third, most MetS components were individually more common in women, but their combination was more frequent in men. This may be due in part to the fact that men with a high cardiovascular risk profile and gout have a higher future risk of type 2 diabetes, independent of other known risk factors [24]. However, in both genders, higher SUA was positively associated with incident hypertension over 9 years of follow-up [26]. Large prospective studies with similar designs and especially identical participant selection criteria could provide explanations for this discrepancy. These studies would also confirm or not whether SUA levels would predict current and future MetS components [6]. Moreover, this difference would tend to blur over time, with a higher proportion of obese women [9].

These data lead us to consider a more drastic management of patients with gout in order to diagnose, prevent, and retard long-term complications of MetS [26]. Thus, morbidity and mortality related to the occurrence of MetS can be reduced [1,2]. Indeed, the increase in SUA over time is associated with an increase in blood pressure and total cholesterol [6]. It is crucial to note that hyperuricemia is not limited to gout [16]. However, with previously published data, our findings do not answer the question of whether hyperuricemia and gout are independent risk factors for MetS [11,16,17] or simply individual components of MetS [17-19]. Pending further studies, hyperuricemia and/or gout are not currently validated components of MetS [15], although they are often present before the onset of MetS components [3,5,16,17,19,20]. This study has several limitations. First, our study was retrospective with many missing data (especially on the lifestyle of our patients). Concerning components of MetS, their frequency was probably underestimated because they were based solely on patients´ statements. Thus, the prevalence of MetS would also be underestimated. Second, it was a monocentric hospital-based study, limiting the number of patients included and also the generalization of the results. Multicenter and even population-based studies are thus expected. Third, the frequency of hypertension and to a lesser extent increased plasma glucose was probably underestimated. This would be mainly related to recall bias. Despite these limitations, the number of patients included in our study makes it one of the largest populations of patients with gout reported in SSA. Besides, our study suggests a probable strong link between gout and MetS in Cameroonians, with specific gender differences. Further studies with a rigorous design are therefore expected to demonstrate the link between gout and MetS, as well as the difference between women and men, while having generalizable data to other SSA populations. One objective will be to provide an answer to the question of whether hyperuricemia and gout should be considered as components of MetS or only as independent risk factors [4,16,17]. In addition, the follow-up of patients with gout in SSA will allow us to appreciate the consequences of the MetS. Moreover, the impact of drug and non-pharmacological preventive as curative measures on reducing mortality related to MetS [2] will also be assessed [17,27].

## Conclusion

In conclusion, MetS and its components are common in newly diagnosed Cameroonian patients with gout. The components of MetS are more common in women, but their combination was more frequent in men. These data should encourage seeking and treating cardiovascular risk factors in patients with gout to reduce morbidity and mortality. Further studies are then required to evaluate these actions in SSA.

### What is known about this topic

- *The relationship between gout and metabolic syndrome remains controversial;*
- *Few studies have specifically investigated the link between metabolic syndrome and gout in sub-Saharan Africa;*
- *Metabolic syndrome is as common in women as in men with gout*.

### What this study adds

- *Metabolic syndrome and its components are common in newly diagnosed gout patients in a sub-Saharan African setting;*
- *The main components of the metabolic syndrome were obesity, hypertension, and diabetes mellitus;*
- *In patients with gout, metabolic syndrome components are more common in women, but their combination was more frequent in men*.
